# Intelligence Instruments Applied to South African School Learners: A Critical Review

**DOI:** 10.3389/fpsyg.2022.853239

**Published:** 2022-06-10

**Authors:** Ilze van der Merwe, Werner de Klerk, Petro Erasmus

**Affiliations:** Community Psychosocial Research, School of Psychosocial Health, North-West University, Potchefstroom, South Africa

**Keywords:** assessment, cross-cultural, ethnic, intelligence, neuropsychology, school learners, South Africa

## Abstract

To establish reliability and validity of formal intelligence assessment instruments in a multicultural and multilingual society such as South Africa, context needs to be taken into consideration and existing psychological intelligence test instruments need to be surveyed carefully for cultural bias. The aim of this critical review of scientific literature was to critically appraise and synthesize data regarding intelligence instruments applied to South African school learners. The search initially yielded 405 studies of which 15 were finally included for thematic analysis. The following three main themes emerged from the included studies: Applicability of intelligence instruments administered to South African school learners; Contextual and demographic influences affecting performance on administered intelligence instruments; and Intellectual measuring instruments related to developmental and cognitive ability levels. It is recommended that the findings of this research study should be considered in the possible development of a strategic guideline to design an intelligence instrument applicable to South African school learners.

## Introduction

The assessment of South African school learners’ cognitive abilities by means of psychological intelligence test instruments has remained controversial, even during the post-apartheid era, where western practices are continuously applied owing to a history of colonialism (Laher and Cockcroft, 2014). Viewed within the context of the country’s unique history, psychologists have conducted irregular intelligence assessment practices, using unsuitable intelligence instruments, due to social and political discourse in South Africa (ZA) during the apartheid era (Laher and Cockcroft, 2014).

Psychologists in ZA have been administering assessment instruments to school learners from diverse, demographic populations, with the challenge of assessing within low-resourced contexts (Laher and Cockcroft, 2017). In addition to limited availability, these available instruments are mostly culturally biased, despite ongoing efforts from the post-apartheid government (Laher and Cockcroft, 2017). In line with the Employment Equity Act 55 (Department of Labour, 1998) as well as the inclusive education policy, psychologists have become aware of the need for linguistic- and cross-culturally relevant assessment practices in the post-apartheid ZA, however, struggle to do so with the lack of ethnically appropriate assessment instruments (Foxcroft et al., 2004). When psychologists assess school learners for diagnostic and scholastic placement reasons, the intelligence test instrument is usually included in the battery of selected assessment instruments. Laher and Cockcroft (2017) stated the imperative need for South African psychologists to become highly resourceful, culturally, and ethically aware by applying emic assessment practices that will be appropriate, applicable, and fair toward the school learners being assessed.

### Legislation Bringing Changes in the New Democratic, Post-apartheid Republic of South Africa

After the first democratic election in 1994, ZA’s new constitution, which included the Bill of Rights, brought about ethnically fair legislations to cater for a multifaceted, diverse society (Meiring, 2007; Shuttleworth-Edwards et al., 2013). This affected the psychological assessment practices in ZA, where assessments had to be administered within various contexts, on multicultural and multilingual populations, using mainly western-normed tests. South African school learners present a vast diversity in its population, providing it a characteristic of various demographic differences. Other than multilinguistic, multi-ethnic and cultural diversities, differences such as quality of education, access to quality resources and services, language proficiency, as well as literacy levels that stemmed from vast socio-economic status (SES) inequalities, have provided multiple challenges to psychological assessment practices and created various assessment environments (Shuttleworth-Edwards, 2016; Laher and Cockcroft, 2017). Thus, in administering intelligence assessment on South African school learners of such a diverse demographic population, presented various questions and challenges.

### Shortage of Appropriate and Culture-Fair Intelligence Instruments to South African School Learners

Consequently, the Human Sciences Research Council (HSRC) investigation of behaviors and needs of test practitioners in ZA, the concerns of outdated, inapplicable psychological tests were raised, accompanied by an urgent appeal to develop psychological tests that are reliable, valid and applicable to the South African population (Foxcroft et al., 2004). Mitchell et al. (2018) mentioned the lack of appropriate and culture-fair intelligence instruments to South African school learners. Laher and Cockcroft (2014, p. 308–309) encouraged assessment and test development practitioners to make active contributions toward “equity and redress” by accommodating “diversity in terms of language, educational background and SES when developing and administering psychological tests”. In order to ensure appropriate and fair psychological assessments of South African school learners from diverse cultures in poorly resourced contexts against a background of social inequality, practitioners need to be “highly resourceful as well as culturally sensitive and ethically aware” (Laher and Cockcroft, 2017, p. 115). Visser and Viviers (2010) stated the obligated necessity of South African psychologists aligning their current practices with the promulgated requirements stated in the Employment Equity Act 55, Section 8 (Department of Labour, 1998). To accomplish this, there exists a need for development of new intelligence assessment instruments and validation of existing ones, which are applicable to all the multifaceted diversity groups found in the South African society. Referring to the South African heterogeneous populations and assessment contexts, Shuttleworth-Edwards (2016) expressed the pressing need to attend to these complexities and to find ways of achieving adequate culture-specific psychological assessment practices.

The choice of individually administered intelligence tests, that South African practitioners assessing school learners presently have, range from the limited and outdated few locally developed intelligence tests for school learners to the western-developed, imported intelligence tests that have been standardized according to South African norms (Shuttleworth-Edwards, 2016; Laher and Cockcroft, 2017). In their edited book: “Psychological assessment practices in South Africa: Research and applications,” Lahar and Cockcroft (2013) listed and discussed various intelligence tests that are administered on school learners in ZA. These included the two local intelligence tests, the 1991 released Senior South African Individual Scale–Revised (SSAIS-R; Van Eeden, 1991) for ages 7–16 years 11 months, and the 1981 released Junior South African Individual Scale (JSAIS; Madge, 1981), assessing children 3–7 years 11 months. The list also included a few imported and locally normed tests of intelligence and cognitive ability, namely the Wechsler Intelligence Scale for Children-fourth edition (WISC-IV; Wechsler, 2003), the Kaufman Assessment Battery for Children-second edition (KABC-II; Kaufman and Kaufman, 2004), and the DAS-Naglieri Cognitive System (CAS; Naglieri and Das, 1997).

### Problem Statement

Lucas (2013) mentioned the lack of locally standardized intelligence tests and speculated that this was due to limited funds and the HSRC changing their role of being the major developing organization. He advised that South African test developers should take hands with international test developers, who have experience in developing high quality tests, and aim to overcome the challenges brought through attempting to develop an assessment instrument for a multicultural society. However, Shuttleworth-Jordan (1996) advocates norming of commonly employed, internationally based cognitive tests for use in the South African context, rather than producing newly devised tests without the benefit of a long history of test refinement through clinical and research practices. Therefore, we need to “think globally, but act locally” (Laher, 2019, p. 2). Based upon above-mentioned statements, there exists a need for designing intelligence instruments that are applicable to the multifaceted, demographically diverse group of school learners found in ZA.

### Goal of the Study

Hence, the aim of this critical review was to critically appraise and synthesize data regarding intelligence instruments applied to South African school learners. We pursued to address the following question: *What does literature state regarding intelligence assessment instruments applied to South African school learners?*

## Method of Investigation

### Research Design

A critical review (De Klerk and Pretorius, 2019) was applied to address the research question, and not a systematic review, due to time constraints as well as the critical purpose of this specific review study. The critical review allowed the researchers to not only search for and describe scientific literature from a variety of resources, but also venture beyond to critically assess the effectiveness and quality of the literature presented (Grant and Booth, 2009).

### Search Approach

A step-by-step literature search was performed in August 2021 using the electronic databases EBSCOhost, Academic Search Complete, E-Journals, PsycArticles, PsycInfo, Sabinet African Journals, ScienceDirect, Google Scholar, as well as South African Theses and Dissertations. A combination of keywords used in the search included cognition terms (“intelligen*” OR “IQ” OR “cognit*” OR “neuropsycholog*”), instrument terms (“test*” OR “assess*” OR “instrument*”), demographic terms (“learner*” OR “schola*” OR child* OR adolescen*) as well as a geographic term (“South Africa*”). Boolean operators such as AND, OR, and NOT were implemented to help clarify the search. Keywords found in searched texts were applied to search additional research studies.

**FIGURE 1:**
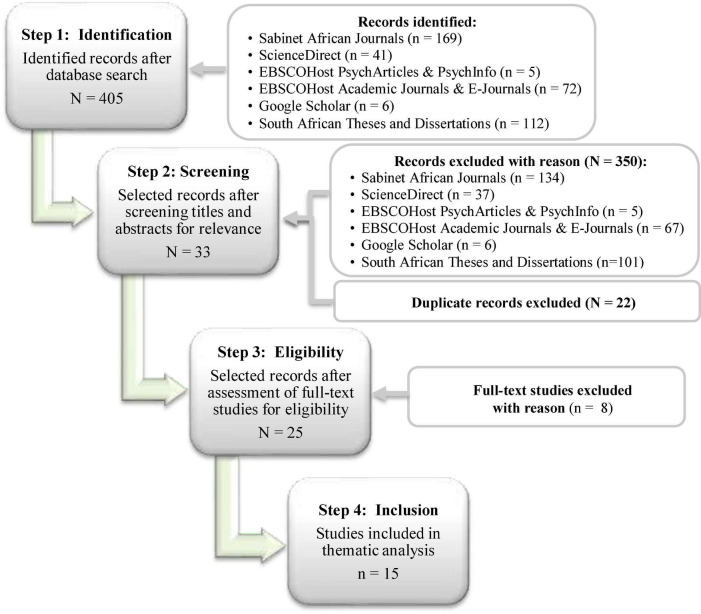
Database search flow chart.

Although this search reviewed peer reviewed works (written in English) from all eras, it predominantly focused on more recent (not older than 10 years) literature works regarding intelligence assessment instruments applied to South African school learners. The search initially yielded 405 studies of which 15 were finally included. Studies were screened and assessed for eligibility by three independent reviewers, namely the primary reviewer (lead author) and co-reviewers (second and third authors), initially by title and abstract, then by full text, according to inclusion and exclusion criteria. Any conflicts were resolved through consensus. Figure 1 presents the four-step search strategy approach as well as inclusion/exclusion criteria. Table 1 provides a summary of the data extracted.

**TABLE 1:** Data extraction table.

Study, aim, tests, and intelligence measured	Sample and SES status	Main results
**Dissertation (Masters of Arts in Psychology: Research): August (2017).** Aim*:* The research aimed to establish psychometric normative screening data based on the Raven’s Colored Progressive Matrices (CPM). Tests*:* The Raven’s CPM. Intelligence measured*:* Both fluid and crystallized intelligences.	Sample: Grade 1–7 school learners (*n* = 388); female (39.95%), male (60.05%); Afrikaans (79%), Xhosa (10%), English (9%); from schools (amount not stated) in the Nelson Mandela Metropolitan Area. SES status*:* School learners with learning barriers.	Results*:* The results of the study indicated that there was a proportional relationship between age and test performance. Gender differences were found where the males outperformed the females. There was a significant difference in the Raven’s CPM test performance with respect to the grade levels of the school learners.
**Minor Dissertation (Magister Educationis in Educational Psychology): Blake (2011).** Aim: To gain an understanding of the school learners’ verbal ability as well as to investigate the quality of the test items on the verbal scale of the translated Sesotho version of the Junior South African Individual Scales (JSAIS). Tests: JSAIS (translated Sesotho version). Intelligence measured: Crystallized intelligence.	Sample: Grade 1 Sesotho school learners (*n* = 29); female (58.62%), male (41.38%); aged 5–7 years, at a primary school in Soweto. The school itself formed part of a greater umbrella research project partnered by the University of Basel Switzerland and the Centre for Education Practice Research at the University of Johannesburg (UJ). SES status: Low-income school learners.	Results: The results show how the Rasch model can provide an in-depth understanding about the functionality and effectiveness of an adapted measure in terms of item difficulty and the participants’ underlying abilities. This study has revealed that the Verbal Intelligence Quotient Eight (VIQ8) scale of the translated JSAIS is a valid and reliable measure, but requires further adaptations in order to make it a suitable instrument for Sesotho Foundation Phase school learners.
**Dissertation (Master of Education in Educational Psychology): Cassoojee (2020).** Aim*:* The main aim of this study was a comparative analysis of the test performance of South African school learners on the indexes/scales of the Wechsler Intelligence Scale for Children - Fifth Edition (WISC-V) and the Kaufman Assessment Battery for Children–Second Edition (KABC-II), so as to determine whether these indexes/scales yield non-significantly different average scores when administered to referred school learners. Tests*:* WISC-V and KABC-II. Intelligence measured*:* Both fluid and crystallized intelligences.	Sample*:* Grades 4–8 school learners (*n* = 50); female (38%), male (62%); 10–14 years old; from a private remedial school in the South of Johannesburg; presenting with specific learning difficulties (SLD), excluding school learners with severe cognitive/developmental delays, severe physical/neurological difficulties, and/or severe emotional or other disorders. SES status: School learners with learning barriers.	Results: The research conclusion was that although these tests show good construct validity, the score discrepancy is significant and they can therefore not be used interchangeably in the South African context.
**Article: De Beer (2005).** Aim: The development of a dynamic test for the measurement of learning potential—the Learning Potential Computerized Adaptive Test (LPCAT). Tests: LPCAT. Intelligence measured: Fluid intelligence.	Sample*:* Grade 9 and Grade 11 school learners (*n* = 2,454); female (49.95%), male (50.05%); Black (49%), White (27%), Colored (24%); schools (*n* = 41); Provinces (*n* = 3). SES status: Diverse socio-economic and educational backgrounds.	*Results:* Development of the LPCAT proved to be a psychometrically sound and practically useful tool for the measurement of learning potential in multicultural contexts.
**Dissertation (Psychiatry: Doctor of Philosophy): Ferrett (2011).** Aim: To ascertain whether cognitive tests developed in settings outside the Western Cape urbanized area have valid application for clinical and research purposes in that area. Tests: The Children’s Color Trails Test (CTTT), Children’s Memory Scales (CMS): Numbers Subtest, CMS: Stories Subtest, CLOX Test, Grooved Pegboard Test (GPT), The Edinburgh Handedness Test (EHI), Maj’s Auditory Learning Test (MAVLT), Rey-Osterrieth Complex Figure Test (ROCFT), Stroop Color-Word Test—Golden Version (SCWT), Tower of London (ToL), Verbal Fluency Tests—Phonemic and Semantic, Wechsler Abbreviated Scale of Intelligence (WASI), Wechsler Intelligence Scale for Children 4th UK Edition (WISC-IV): Coding Subtest. Intelligence measured: Both fluid and crystallized intelligences.	*Sample:* Afrikaans (43.3%)–and English-speaking (56.7%) adolescents (*n* = 215); female (54.4%), male (45.6%); Colored (68.8%), White (31.2%); aged between 12 and 15 years; with between 6 and 10 years of completed education; recruited from schools (*n* = 47) in the greater Cape Town metropolitan region. SES status*:* Diverse socio-economic and educational backgrounds.	Results*:* This dissertation provided (1) methodological guidelines to assessing, adapting, translating, and norming cognitive tests, and (2) stratified norms for a selection of cognitive tests, for a narrowly defined population.
**Article: Jansen and Greenop (2008).** Aim: The study used the K-ABC in a longitudinal application in order to explore the robustness of the factor structure of the K-ABC for the same group of children at two developmental points (5 and 10 years) in time. Tests: K-ABC. Intelligence measured: Both fluid and crystallized intelligences.	Sample*:* Children living in Soweto (*n* = 199); at 5 and 10 years; female (51%), male (49%); isiZulu (42%), Sotho (26%) Tswana (16%), Xhosa (6%), Venda (6%), and Tsonga (4%). SES status*:* Changes in the economic circumstances were reflected at the two time stages. At 5 years, 73% lived in shared housing, at 10 years 41% lived in shared housing.	Results*:* Employing the K-ABC as a theory driven assessment tool with a South African population is feasible and useful.
**Article: Jinabhai et al. (2004).** Aim: This study reports on the application and interpretation of a selected battery of mental ability tests among isiZulu school learners and the methodological and analytical issues that need to be addressed. Tests: CPM, Auditory Verbal Learning Test (AVLT), Symbol Digit Modalities Test (SDMT), Young’s Group Mathematics Test (GMT). Intelligence measured: Both fluid and crystallized intelligences.	Sample*:* IsiZulu Grade 3 school learners (*n* = 806); female (43.9%), male (56.1%); ages 8–11; rural primary schools (*n* = 11); Vulamehlo Magisterial District, Kwazulu-Natal. SES status*:* Rural community.	Results*:* Significant gender differences were found in the test scores, and the mean scores of isiZulu school learners in this study were lower than those reported in other studies.
**Article: Levert and Jansen (2001).** Aim: Explores Piagetian and Lurian approaches to the assessment of neurocognitive processes of previously disadvantaged Black school learners attending English medium schools in the Johannesburg area. Tests: Home Screening Questionnaire (HSQ), Clinical Neuropsychological Evaluation Instrument, Piagetian Tasks, and Draw-a-Person (DAP) Test. Intelligence measured: Fluid intelligence.	Sample*:* Historically disadvantaged Black school learners (*n* = 50) in English medium schools (*n* = 3) in Johannesburg at 7 years old; with learning problems (*n* = 27) and without learning problems (*n* = 23); female (52%), male (48%); Southern Sotho or Northern Sotho (57%), isiZulu (26%), and Venda and Xhosa (13%). SES status*:* Diverse socio-economic and educational backgrounds.	Results*:* Piagetian and Lurian approaches can offer greater insight into South African school learners’ cognitive levels and task performance.
**Minor Dissertation (Magister Educationis in Educational Psychology): Mawila (2012).** Aim*:* To investigate the quality of the test items on the sub-scales of the performance subtest of the translated Sesotho version of the JSAIS (GIQ-8). Tests*:* JSAIS (translated Sesotho version). Intelligence measured*:* Fluid intelligence.	Sample*:* Sesotho Grade 1 school learners (*n* = 42) in a school in Soweto, who come from linguistically, culturally and socio-economically diverse backgrounds. Female and male were not specified. SES status*:* Diverse socio-economic and educational backgrounds.	Results*:* Results suggest the Form Board subtest of the JSAIS (GIQ-8) is not a valid measure for the Sesotho participants whereas Absurdities A and B is likely to be a valid measure for this population. However, further adaptation that include omittance of redundant items and redesigning of pictures, should make it a suitable instrument for participants in this particular context.
**Article: Mitchell et al. (2018).** Aim: To establish the reliability and validity of the K-ABC-II. Tests: K-ABC-II (translated isiZulu version). Intelligence measured: Both fluid and crystallized intelligences.	Sample*:* HIV-uninfected primary school-aged children (n = 376) in rural Northern KwaZulu-Natal between 7 and 11 years old; female (65%), male (35%). SES status: Rural community.	Results*:* With translation and the inclusion of supplementary sub-tests, the K-ABC-II has good reliability and maintains its original structure when used in this context.
**Minor Dissertation (Magister Educationis in Educational Psychology): Naicker (2013).** Aim*:* To apply a Rasch Analysis to the Numerical Intelligence Quotient Eight (NUM Scale) of an isiZulu translation of the JSAIS to determine the quality of the items in relation to the ability of participants in the sample group. Tests*:* JSAIS (translated isiZulu version). Intelligence measured*:* Both fluid and crystallized intelligences.	*Sample:* Grade 1 isiZulu school learners (*n* = 34); female (47.06%), male (52.94%); at a school in Soweto, an urban area within Johannesburg with predominance of black persons, where many parts are socio-economically disadvantaged and speaking varying dialects. The longitudinal research project is a collaborative effort between the University of Basel, Switzerland and the Centre for Education Practice Research (CEPR) at the University of Johannesburg (UJ). SES status*:* Socio-economically disadvantaged.	Results*:* Results of the study show that the data generated varied in its fit of the Rasch Model. Results also confirmed that the numerical subtests of the Number and Quantity are valid measures of the construct for which it was designed; but further studies on the isiZulu translated JSAIS are recommended, with larger samples, reduced cultural loading on items, as well as consideration of found misfitted items, item difficulty, and item overlap.
**Article: Reid et al. (2002).** Aim: Whether results obtained with the PASS (Planning, Attention, Simultaneous and Successive) model of intelligence could provide insight into the cognitive functioning of South African school learners. Tests: Cognitive Assessment System (CAS), Woodcock Diagnostic Reading Battery (WDRB), and school marks. Intelligence measured: Both fluid and crystallized intelligences.	Sample*:* Grade 6 Black and English second language school learners (*n* = 32) at an urban public school in a Northern suburb of Johannesburg. Female and male not specified. SES status*:* Not specified.	Results*:* The results indicated that the PASS model for intelligence correlates with reading and scholastic achievement in the South African context.
**Book Chapter: Shuttleworth-Edwards et al. (2013).** Aim: Investigating the United Kingdom-version WISC-IV test performance with the objective of producing comparative normative indications for the ten core subtest scores, four Index scores, and the Full Scale Intelligence Quotient (FSIQ) score that could be utilized in typical clinical situations as they currently apply in the South African context. Tests: WISC-IV-UK. Intelligence measured: Both fluid and crystallized intelligences.	Sample*:* Grade 7 school learners (N = 69); female (47.83%), male (52.17%); 12–13 years old; White (34.78%), Black (34.78%), Colored (30.44%); Afrikaans (47.83%), Xhosa (34.78%), English (17.39%); in Makhanda (previously Grahamstown), Ggeberha (previously Port Elizabeth), and Cape Town. SES status*:* Diverse socio-economic and educational backgrounds.	Results*:* The data in respect of the WISC-IV, while also in respect of small sample numbers, gain validity in that the sample is well-stratified for the relevant socio-cultural variables. Further, clear replication of the adult findings in this child-oriented research, of a downward continuum of IQ test performance in association with poorer quality of education rather than ethnicity *per se*, provides cross-validation for both the adult and child research probes.
**Minor Dissertation (Magister Educationis in Educational Psychology): Teixeira (2011).** Aim*:* To apply a Rasch Analysis to the VIQ-8 scale of an isiZulu translation of the JSAIS to determine whether it would be a reliable and valid means of assessing the verbal cognitive functioning and development of school learners at a school in the South African context. Tests*:* JSAIS (translated isiZulu version). Intelligence measured*:* Crystallized intelligence.	Sample*:* Grade 1 isiZulu school learners (*n* = 26); female (57.69%), male (42.31%); ages 5–7 years old; at a school in Soweto. Research was conducted at the school under the auspices of the Soweto Panel Research Program. SES status: Not specified.	Results*:* The analysis of the results suggested that on the whole the three sub-tests fitted the Rasch Model well.
**Dissertation (Master of Social Science in Psychology): Van Wyhe (2012).** Aim*:* To describe the development of a culturally and linguistically adapted version of the WASI suitable for use in English and Afrikaans 12–15 years old first-language speakers. Tests*:* Demographic and Socio-economic Questionnaire, Marin Acculturation Scale (MAS), and WASI-SA (English and translated Afrikaans versions). Intelligence measured: Both fluid and crystallized intelligence.	Sample*:* School learners (*n* = 200); female (53%), male (47%); 12–15 years old; Afrikaans (53%), English (47%); Western Cape. SES status*:* Diverse socio-economic and educational backgrounds.	Results*:* This study provides valuable guidelines for collection and use of normative data for research and clinical purposes in ZA.

### Procedure

This research study (a critical review) received approval from the Health Research Ethics Committee (NWU-00191-21-A1) of the Faculty of Health Sciences, North-West University (NWU), ZA. The primary reviewer (lead author) performed an independent search, while the second reviewer (second author) monitored the review process by acting as co-analyst of extracted data. The seven-step critical review guideline, as suggested by De Klerk and Pretorius (2019), was followed to ensure optimal data gathering. The PRISMA and PICO approaches were not implemented in this critical review study, rather we implemented a simple analytical framework (Grant and Booth, 2009)—Search, Appraisal, Synthesis and Analysis (SALSA)—for the main purpose of this critical review study.

### Data Analysis

The thematic data analysis method was applied to analyze the extracted data (Clarke and Braun, 2018). First, the primary reviewer (lead author) familiarized herself with the data by reading and re-reading the identified literature. Second, the lead author identified codes for use in creating themes by sorting the codes according to a pattern. Third, we (lead author and two co-authors) defined and named the identified themes for the findings statement. Lastly, we produced the written report (this critical review article).

## Findings and Discussion

The following three main themes emerged from the included studies: (i) Applicability of intelligence instruments administered to South African school learners; (ii) Contextual and demographic influences affecting performance on administered intelligence instruments; and (iii) Intellectual measuring instruments related to developmental and cognitive ability levels. Table 1 provides a summary of included studies. We discuss these themes below.

### Theme 1: Applicability of Intelligence Instruments Administered to South African School Learners

It was evident from all the studies that intelligence assessment instruments administered to South African school learners from diverse, demographic populations need to be applicable to that particular population subgroup, in order to be valid, reliable, and fair (Levert and Jansen, 2001; Reid et al., 2002; Jinabhai et al., 2004; De Beer, 2005; Jansen and Greenop, 2008; Ferrett, 2011; Teixeira, 2011; Mawila, 2012; Van Wyhe, 2012; Shuttleworth-Edwards et al., 2013; August, 2017; Mitchell et al., 2018). When administering imported western-developed intelligence instruments to South African school learners, appropriate and updated norms need to be applied, based on continuous normative studies conducted on an equivalent population group and within a similar context as the testees (Ferrett, 2011; Van Wyhe, 2012; Shuttleworth-Edwards et al., 2013; August, 2017). The international theory driven K-ABC and K-ABC-II have proven to be feasible, valid, reliable, and appropriate intelligence assessment instruments when administered to multicultural South African school learners (Jansen and Greenop, 2008; Mitchell et al., 2018). The research study by Mitchell et al. (2018) found the K-ABC-II to have good construct validity and reliability when applied to a South African school learner sample, provided that it is translated, the battery maintains its original structure (allowing insight into performance on various cognitive domains), and applicable supplementary subtests are included (to strengthen the measurement of the Planning subscale) in the assessment.

A few attempts have been made to investigate, adapt, update, and norm the locally developed intelligence test JSAIS, however, not without significant challenges. In the research studies conducted by students at the University of Johannesburg, the researchers respectively administered Sesotho-translated and isiZulu-translated versions of the JSAIS to South African school learners and employed the Rasch model to determine item accuracy (do items measure what they are intended to measure) and applicability (Blake, 2011; Teixeira, 2011; Mawila, 2012; Naicker, 2013). Mawila (2012), who administered the Sesotho-translated version of the JSAIS Performance subscales found that it could be a valid measure for South African school learners if foreign, redundant, and outdated items are adapted and translation challenges (many concepts could for instance not be accurately translated) are addressed. Teixeira (2011) found the isiZulu-translated JSAIS Verbal subscales to be a valuable intelligence assessment instrument as it still held sound psychometric properties, however, needed to be updated, adapted, and re-normed to adequately fit the South African school learner population. Similar findings from the Sesotho-translated JSAIS Verbal subscales disclosed it as a valid and reliable intelligence measure, however, with the recommendation of needed adaptations to make it suitable for the South African school learners being assessed (Blake, 2011). Naicker (2013) investigated the Numerical scales of an isiZulu-translated JSAIS and found the included numerical subtests to be valid measures of the constructs for which they were designed, but further similar studies are recommended, with larger samples, reduced cultural loading on items, as well as consideration of found misfitted items, item difficulty, and item overlap. Moloi (2014) conducted a research study investigating the quality of various test items found in the isiZulu-translated JSAIS Global Intelligence scales, with the purpose of determining how they can be improved to be an appropriate measure for South African school learners. Findings included themes of language and numerical issues derived from test translation as well as a need to revise and update pictures and other visual data to be more relatable and familiar to the group being tested (Moloi, 2014).

In reaction to the challenges of assessing South African school learners from multicultural contexts, the LPCAT was locally developed and proved to be a fair and psychometrically sound non-verbal intelligence test, as it was developed and standardized using multicultural samples and yields information over a wide range of abilities (De Beer, 2005).

### Theme 2: Contextual and Demographic Influences Affecting Performance on Administered Intelligence Instruments

Some reviewed studies noted significant differences on intelligence assessment results among various South African school learner subgroups, and related performance differences to contextual and demographic influences (Levert and Jansen, 2001; Jinabhai et al., 2004; De Beer, 2005; Ferrett, 2011; Van Wyhe, 2012; Shuttleworth-Edwards et al., 2013; August, 2017; Mitchell et al., 2018). Some studies found that English school learners from an advantaged educational background performed at the highest level of all the subgroups tested (Ferrett, 2011; Van Wyhe, 2012; Shuttleworth-Edwards et al., 2013). The normative study by Shuttleworth-Edwards et al. (2013) administering the WISC-IV-UK to school learners from advantaged and disadvantaged quality of education revealed the following findings: the advantaged (private schooling) English White subgroup received the highest mean scores, followed by the advantaged (private and Model C schooling) Black Xhosa subgroup as well as the advantaged (non-private, Model C schooling) White Afrikaans subgroup, and subsequently the disadvantaged education subgroups performing the lowest with the Black Xhosa scoring within borderline range and the Colored Afrikaans within the extremely low range. The researchers concluded by stating that the WISC-IV performance revealed a clear downward continuum of intelligence performance in relation to poorer quality of education, rather than ethnicity, and cross-validated this finding with other similar South African research studies on Wechsler intelligence scales [see Zindi (1994), Brown (1998), and Shuttleworth-Edwards et al. (2004)]. Van Wyhe (2012) found South African school learners from disadvantaged quality of education performing more poorly than the school learner subgroup from advantaged quality of education across all subtests and index scores of the WASI, regardless of home language. A normative study by August (2017) found an upward continuum of intelligence performance on the CPM in relation to increased age/grade levels as well as gender differences in test performance. These findings were in alignment with other similar local and international normative studies conducted on subgroups with differing geographic and demographic features [see Raven et al. (1990), Knoetze et al. (2005), and Linstrom et al. (2008)].

A trend of South African school learners performing more often in a lower range compared to international norms, were identified by many of the studies reviewed (Jinabhai et al., 2004; Ferrett, 2011; Van Wyhe, 2012; Shuttleworth-Edwards et al., 2013; August, 2017; Mitchell et al., 2018). These studies attributed the inferior performance to contextual and demographic differences, specifically language differences (school learners being assessed in a second/third language or receiving translated test instructions, where certain concepts could be misinterpreted or “lost in translation”) and disadvantaged quality of education which resulted from previous apartheid policies and inferior SES backgrounds (Jinabhai et al., 2004; Ferrett, 2011; Van Wyhe, 2012; Shuttleworth-Edwards et al., 2013; August, 2017; Mitchell et al., 2018). Based on findings from a study administering a selected battery of imported intellectual tests to South African school learners from rural disadvantaged primary schools and referring to a statement made by Spreen and Strauss (1991), Jinabhai et al. (2004) reported that the lower mean mental ability scores found, compared with other local and international studies’ performances, may be invalid comparisons as the various school learner subgroups have educational and cultural differences. Hypotheses for lower performances included disadvantaged quality of education, possible lack of familiarity with psychometric testing tasks, as well as non-verbal tests that could be culturally loaded with unfamiliar concepts (Jinabhai et al., 2004). Van Wyhe (2012) reasoned that their study’s findings of lower intelligence performance of South African school learners with disadvantaged schooling, compared to American counterparts, could be due to disadvantaged education and experienced “language conflict” (bilingualism and multilingualism possibly affecting cognitive and linguistic abilities).

Some reviewed studies found that English school learners from an advantaged educational background performed at the same level or even slightly better than their American counterparts (Van Wyhe, 2012; Shuttleworth-Edwards et al., 2013). Mitchell et al. (2018) researched the intellectual performance of South African school learners by administering the K-ABC-II and found lower mean scores on the Simultaneous and Planning subscales as opposed to the Learning and Sequential subscales. The researchers gave probable explanations to this trend by identifying possible test-related factors of timed-points and test content where school learners’ test approach and performances could differ depending on their socio-demographic characteristics, SES, as well as test and school exposure (Mitchell et al., 2018). Referring to the tendency to link concepts of “intelligence” and “responsibility” in African contexts, the researchers attributed timed points lost to school learners probably taking their time to consider tasks carefully (acting responsibly) instead of completing them swiftly, and noted possible bias on test content such as use of certain shapes, forms or pictures that could be unfamiliar due to lack of previous exposure (Mitchell et al., 2018).

### Theme 3: Intellectual Measuring Instruments Related to Developmental and Cognitive Ability Levels

The South African developed LPCAT proved to be a psychometrically sound, culturally fair, time-efficient, and practically useful intelligence instrument which lessens the impact of SES, cultural or educational influences of persons from multicultural contexts by measuring non-verbal fluid intelligence (De Beer, 2005). This dynamic assessment instrument was developed, standardized and validated using multicultural samples, and in applying item response theory principles (where item parameters do not depend on the ability level of the testees, but are a property of the item) and computerized adaptive testing technology allows for more accurate measurement of difference scores, making it very applicable to South African school learners (De Beer, 2005).

The research study by Mitchell et al. (2018) administering the K-ABC-II to South African school learners, found that they received lower means on the Simultaneous and Planning subscales as opposed to the Learning and Sequential subscales; researchers referred to a similar finding which occurred in another research study on an Indian population [Malda et al., 2008, as cited in Mitchell et al. (2018)]. In addition to identified test-related factors, researchers hypothesized skilled-related factors as possible attributes to the lowered performance, which included underdeveloped cognitive executive functioning skills. Researchers, however, reflected on the study’s limitation of not examining the effect that environmental factors could have had on the emergence of executive functioning skills or how these possibly influenced the school learners’ performance (Mitchell et al., 2018). Based on the study findings by Mitchell et al. (2018) noting that the K-ABC-II had high reliability and construct validity when administered in the South African context, Cassoojee (2020) conducted a comparative analysis of South African school learner test performance on the theoretically similar indexes/scales of the United Kingdom normed WISC-V and K-ABC-II. The findings indicated good construct validity with strong correlations between most of the similar indexes and scales; specifically, a strong correlation between the Working Memory Index (WMI) and the Knowledge Scale (Gc) suggested an important association between working memory and crystalized (prior learning) intelligence (Cassoojee, 2020).

Based on the Piaget-inspired PASS (Planning, Attention, Simultaneous, and Successive) model, the CAS was administered to South African school learners which revealed findings of a significant correlation between the school learners’ cognitive processes assessed by the CAS, as well as their reading ability (measured by the WDRB) and school marks (Reid et al., 2002). Findings of CAS as a valid and fair intelligence instrument when applied to South African school learners seemed promising but was not confirmed in this research study by Reid et al. (2002); nevertheless, the findings provided pivotal insight into understanding the school learners’ cognitive processes involved during intelligence assessment which were directly linked to the success or failure of their reading performance (a required scholastic skill) as well as school performance. Levert and Jansen (2001) introduced Piagetian tasks (measuring developmental cognitive levels) and Lurian tasks of reading and writing, arithmetic, language, visual, mnestic, intelligence, and motor functioning (similar to required formal scholastic skilled tasks) to assess the intelligence of two subgroups, a Learning Problems (LP) group and a No Learning Problems (NLP) group. The LP group was found to perform at Piaget’s Preoperational stage of cognitive development (with non-attained scores on tasks of conservation and seriation, but intermediate level mean scores on classification tasks) which related significantly to their performance on Lurian tasks (Levert and Jansen, 2001). Levert and Jansen (2001) found the NLP functioning at higher maturation levels compared to the LP group, namely within the beginning stages of the Piagetian Concrete Operational stage (with intermediate level mean scores for tasks of seriation, classification, and conservation), which linked to the mastery of most Lucian tasks (except for presenting slight difficulties in expressive language, mnestic processes, and intelligence tasks).

## Implication for Intelligence Assessment Within the South African Multi-Diverse Context

In order to keep updated and suitable intelligence tests for all South African school learner subgroups, normative studies need to continue across the whole country (Levert and Jansen, 2001; Jinabhai et al., 2004; De Beer, 2005; Ferrett, 2011; Van Wyhe, 2012; Shuttleworth-Edwards et al., 2013; August, 2017; Mitchell et al., 2018).

Most of the subtests in the locally developed JSAIS were found to be valid intellectual assessment measures on South African school learner subgroups, but with the recommendation of refinement in the translation efforts as well as updating, adapting, and standardizing this instrument with norms that are representative of the South African population (Blake, 2011; Teixeira, 2011; Mawila, 2012; Naicker, 2013; Moloi, 2014). Moloi (2014) gave various examples of inapplicable test item content that needs modification. Such as translation errors or difficulties caused by vocabulary used out of context (changing the meaning of the word), use of terminology other than the school learner’s dialect, use of terms not applied in school (e.g., mathematical terms), difficulty when translating certain items such as certain riddles or the “rhymes with” questions, or total untranslatability. Some questions and illustrations were found not to be user-friendly, outdated, unfamiliar (from a western context), not age appropriate (e.g., item 32 could be too detailed for young children), ethnically biased (e.g., Performance scale item 32 with a picture of a boat; or Verbal scale item 27 stating that “Dogs are tame and wolves are.?” could be misleading where dogs were perceived by many participants as things to be afraid of). These findings were confirmed by Theron (2013) who suggested that certain concepts in the JSAIS are foreign to school learners who differ in their background, language, or ethnicity with examples such as: using the word “freckles” in the Vocabulary subtest to a non-White child; saying phrases like “in the veld,” “in a vase” “in the dining room” in the Story Memory subtest to a child with a different cultural heritage. In addition, caution was drawn toward certain questions, words, or phrases found in the JSAIS subtests Ready Knowledge, Word Association, or Vocabulary that could trigger memories of loss and/or crime, as many South Africans have experienced, that could provide inaccurate findings (Theron, 2013). Foxcroft (2011, p. 12) noted one challenge when aspiring to translate tests into an indigenous language, is finding a bilingual expert who does not simply translate on a word-for-word basis, but one who comprehends the “idiosyncrasies and subtle nuances of both languages as well as the social meanings attached to words and phrases.” Moloi (2014) recommended both local educators (speaking the same language as the tested subgroup) and psychologists review and adapt the content of the intelligence test instrument for validity, language applicability, and cultural appropriateness before administering it to the South African school learner subgroup. Efforts of suitable test adaptation and standardization would need, however, a lot of time and resources to conduct.

The time-efficiency and applicability of the LPCAT instrument (De Beer, 2005) could address the lack of resources for intellectual testing in ZA and reach more school learners to develop sufficient normative data. With the purpose of assessing school learners fairly, especially those school learners from a deprived quality educational background, it is recommended that intellectual instruments such as the LPCAT be selected, since it applies fluid intelligence as opposed to crystallized intelligence; fluid intelligence assesses the reasoning ability of novel problems and is not dependent on language proficiency or prior school learning, whereas, crystallized intelligence is based on acquired knowledge (De Beer, 2005). Imported intelligence tests that only contain non-verbal subtests, have been administered to various South African school learner subgroups, in the hope to eliminate language and cultural bias; although excluding many potential bias challenges, non-verbal tests still use language and illustrations that could have cultural elements that may result in certain test items to be bias and inapplicable to the school learners tested (Jinabhai et al., 2004; Ferrett, 2011; Van Wyhe, 2012; August, 2017). Internationally developed intelligence instruments containing various indexes/scales such as the K-ABC-II and WISC-V, were developed on a theoretical base of cognitive process abilities including Spearman’s g-theory, the Cattel-Horn-Carrol theory and the PASS theory model (Kaufman et al., 2016; Mitchell et al., 2018; Cassoojee, 2020). Theory underlying intelligence instruments allows for insight into the different domains of cognition (Mitchell et al., 2018). A cognitive profile in combination with results from linguistic and culturally fair intelligence instruments, should allow practitioners to make more accurate diagnoses and develop more suitable intervention strategies (such as school placements or special needs educational plans) for South African school learners (Levert and Jansen, 2001; Reid et al., 2002; Jinabhai et al., 2004; Ferrett, 2011; Van Wyhe, 2012; Shuttleworth-Edwards et al., 2013; August, 2017).

Viewing intelligence assessment within the unique, multi-diverse ZA context is challenging, but also serves as an opportunity to advance intelligence instruments and their application in practice not only in ZA, but also globally. When used correctly the intelligence assessment is an efficient tool, yet it can be destructive when abused. Besides ensuring that the instrument itself is valid, reliable and optimally designed, the application and use of the instrument should be considered equally important. It is suggested that a thorough and user-friendly manual accompany the intelligence instrument at all times. The manual should cover topics including the specific purpose of the instrument, implied risks and safety measures, client and context limitations, as well as scope of the instrument. Detailed examples of case studies should outline the correct assessment procedure and possible pitfalls. Proper procedure is built on the four pillars of assessment as suggested by Sattler (2001) to triangulate findings. Official training of test users (and timely refresher courses) remains a key requirement to administer the instrument responsibly and optimally.

## Limitations and Recommendations

While, a critical review does serve to cluster the literature on a topic, the interpretative elements are necessarily subjective and the resulting product is the starting point for further evaluation, not an endpoint in itself (Grant and Booth, 2009). It is recommended that the findings of this research study (a critical review) should be considered in the possible development of a strategic guideline to design an intelligence instrument applicable to South African school learners.

## Conclusion

Increased research has been undertaken in the psychometric field on local and national normative studies regarding various assessment measures in ZA (August, 2017). However, contextual factors could influence the applicability of the test instrument, as well as the school learners’ test-taking skills/familiarity and prior learning (crystalized intelligence), and/or the school learners’ cognitive abilities (fluid intelligence and other constructs of intelligence). Practitioners will need to consider this when developing or selecting intelligence test instruments to administer to specific South African school learner subgroups, as well as when interpreting test results for diagnosis and intervention purposes. As previously mentioned, a thorough history and insight of the school learners’ ethnic, quality of school, and SES background in combination with a cognitive profile drawn from developmental, linguistic, and culturally fair intelligence instruments, should allow practitioners to make more accurate diagnoses and develop more suitable intervention strategies for South African school learners.

## Author Contributions

All authors listed have made a substantial, direct, and intellectual contribution to the work, and approved it for publication.

## Conflict of Interest

The authors declare that the research was conducted in the absence of any commercial or financial relationships that could be construed as a potential conflict of interest.

## Publisher’s Note

All claims expressed in this article are solely those of the authors and do not necessarily represent those of their affiliated organizations, or those of the publisher, the editors and the reviewers. Any product that may be evaluated in this article, or claim that may be made by its manufacturer, is not guaranteed or endorsed by the publisher.
